# Dermal phospho-alpha-synuclein deposits confirm REM sleep behaviour disorder as prodromal Parkinson’s disease

**DOI:** 10.1007/s00401-017-1684-z

**Published:** 2017-02-08

**Authors:** Kathrin Doppler, Hanna-Maria Jentschke, Lena Schulmeyer, David Vadasz, Annette Janzen, Markus Luster, Helmut Höffken, Geert Mayer, Joachim Brumberg, Jan Booij, Thomas Musacchio, Stephan Klebe, Elisabeth Sittig-Wiegand, Jens Volkmann, Claudia Sommer, Wolfgang H. Oertel

**Affiliations:** 1 Department of Neurology, University Hospital Würzburg, Josef-Schneider-Str. 11, 97080 Würzburg, Germany; 2 Department of Neurology, Philipps University Marburg, Baldingerstr., 35043 Marburg, Germany; 3 Department of Nuclear Medicine, Philipps University Marburg, Baldingerstr., 35043 Marburg, Germany; 4 Department of Nuclear Medicine, University Hospital Würzburg, Oberdürrbacher Str. 6, 97080 Würzburg, Germany; 5 Department of Nuclear Medicine, Academic Medical Centre, University of Amsterdam, Meibergdreef 9, 1105 Amsterdam, The Netherlands; 6 Department of Neurology, University Hospital Freiburg, Breisacher Str. 64, 79106 Freiburg, Germany; 7 Institute for Neurogenomics, Helmholtz Institute for Health and Environment, Ingolstädter Landstr. 1, 85764 Neuherberg, Germany

**Keywords:** REM sleep behaviour disorder, Parkinson’s disease, Alpha-synuclein, Skin biopsy, FP-CIT-SPECT

## Abstract

Phosphorylated alpha-synuclein (p-alpha-syn) deposits, one of the neuropathological hallmarks of Parkinson’s disease (PD), have recently been detected in dermal nerve fibres in PD patients with good specificity and sensitivity. Here, we studied whether p-alpha-syn may serve as a biomarker in patients with a high risk of developing PD, such as those with REM sleep behaviour disorder (RBD). We compared the presence and distribution of p-alpha-syn deposits in dermal nerve fibres in 18 patients with RBD, 25 patients with early PD and 20 normal controls. Skin biopsy was taken at C7, Th10, and the upper and lower leg. Presynaptic dopamine transporter imaging using FP-CIT-SPECT was performed in all patients with RBD and in 11 patients with PD. All RBD patients underwent olfactory function testing. The likelihood ratio (LR) for prodromal PD was calculated for each patient based on published research criteria. Skin serial sections were assessed by double-immunofluorescence labelling with antibodies to pSer129-alpha-syn under blinded conditions. P-alpha-syn was visualized in 10/18 patients with RBD (sensitivity of 55.6%) and in 20/25 early PD patients (sensitivity of 80%) but in none of the controls (specificity of 100%). The percentage of dermal structures innervated by p-alpha-syn-positive fibres was negatively correlated with dopamine transporter binding in the FP-CIT-SPECT (*ρ* = −0.377, *p* = 0.048), with olfactory function (*ρ* = −0.668, *p* = 0.002), and positively correlated with the total LR for RBD to present prodromal PD (*ρ* = 0.531, *p* = 0.023). Dermal p-alpha-syn can be considered a peripheral histopathological marker of synucleinopathy and can be detected in a subgroup of RBD patients presumably representing prodromal PD. Dermal p-alpha-syn is detectable in RBD patients without PD motor symptoms, thereby stratifying a patient group that is of great interest for clinical trials testing disease-modifying drugs.

**Electronic supplementary material:**

The online version of this article (doi:10.1007/s00401-017-1684-z) contains supplementary material, which is available to authorized users.

## Introduction

Parkinson’s disease (PD), like other neurodegenerative disorders, does not start suddenly, but progresses through early “prodromal” stages, in which the defining motor symptoms have not yet emerged. This poses an important obstacle in clinical research for disease-modifying therapies, as PD only becomes clinically obvious when patients have already lost 80% of dopaminergic nerve terminal function in the posterior putamen [24, 27] and an estimated 30–50% of nigral dopaminergic neurons have died [11]. A definite diagnosis of PD is based on pathologic confirmation of alpha-synuclein containing Lewy bodies and dopamine neuronal loss in the substantia nigra pars compacta with a pre-mortem history of Parkinsonism, but this “gold standard” is only established after death.

We and others have recently proposed that histopathological confirmation of PD may be advanced by pre-mortem biopsies demonstrating phosphorylated Ser129-alpha-synuclein (p-alpha-syn) deposition within neurons and neurites of the enteric nervous system, the submandibular gland, or the skin, closely resembling the central nervous system pathology [5, 6, 8, 20, 26, 39]. Skin biopsies are particularly easy to perform. Peripheral p-alpha-syn pathology within dermal nerve fibres has proven to have high sensitivity and specificity in differentiating PD from tauopathies or other atypical forms of Parkinsonism [6, 8, 10].

So far, dermal p-alpha-syn deposition has only been studied in patients with clinically manifest, i.e. motor PD. It is unknown if dermal nerve fibres become involved in the disease process during premotor stages and how they relate to other markers of prodromal PD. In this study, we address the potential value of dermal p-alpha-syn deposition as a premotor histopathological biomarker of PD by evaluating skin biopsies in patients with REM sleep behaviour disorder (RBD), a well-established clinical non-motor risk marker of PD [16, 18]. Patients with RBD have an approximately 85% risk of conversion into a clinically manifest alpha-synucleinopathy [dementia with Lewy bodies (DLB), PD or multiple system atrophy (MSA)] within 15-20 years, among which PD and DLB are the most frequent [16, 18, 23]. Accordingly, neuropathological studies of patients with RBD have revealed the presence of Lewy bodies in the brainstem [3, 16, 37].

By combining a diagnosis of RBD with other clinical non-motor signs of neurodegenerative alpha-synucleinopathies, such as olfactory dysfunction and neuroimaging of impaired dopamine transporter ligand binding (i.e. 123I-*N*-ω-fluoropropyl-2β-carbomethoxy-3β-(4-iodophenyl)tropane single photon emission computed tomography, FP-CIT-SPECT), one can define a subgroup of patients with a high probability of conversion to motor Parkinsonism within a few years [17, 22]. Using probability methodology, research criteria for prodromal PD have been proposed as a hypothetical concept by the International Parkinson and Movement Disorder Society (MDS) [2] for validation and updating in future studies, as recently employed in a cohort of elderly people [21].

In this case control study, we compare the presence and extent of dermal p-alpha-syn depositions in patients with a high risk of prodromal PD, i.e. RBD patients, with early motor stages of PD and healthy controls.

## Materials and methods

The study was approved by the ethic’s committees of the Universities of Würzburg and Marburg, Germany. All patients and controls gave written informed consent to participate.

### Patients and controls

20 patients with idiopathic RBD who attended the Department of Neurology, University Marburg, Marburg, Germany, for diagnostic work-up were prospectively recruited between December 2014 and July 2016. Two of these patients were later excluded, one because of suspected secondary (medication-induced) RBD that completely remitted, and one who turned out to have developed MSA at the time of skin biopsy. Consequently, 18 patients were finally included in our study. 25 patients with PD Hoehn and Yahr (H&Y) stages 1 and 2 [14] were prospectively recruited from the Departments of Neurology at the University Hospitals Marburg and Würzburg during the same time period. Skin biopsies were obtained from the patients’ spouses without clinical symptoms of PD or RBD as controls for p-alpha-syn detection (*n* = 20). RBD patients were diagnosed by medical history followed by video-polysomnography according to the consensus criteria of the International RBD Study Group [31] and to the International Classification of Sleep Disorders (version 3) [25]. Patients with PD were diagnosed according to the United Kingdom Brain Bank criteria [15] and staged according to Hoehn and Yahr [14]. The severity of motor symptoms was assessed in RBD and PD patients using the Unified Parkinson’s Disease Rating Scale (UPDRS) part 3 [13] and non-motor symptoms were measured by the German version of the non-motor symptom scale [36]. The RBD screening questionnaire (RBD-SQ) was employed for the subjective reporting of RBD related symptoms in the RBD group [34]. The Beck Depression Inventory (BDI) was applied for assessment of depressive symptoms [1], the Montreal Cognitive Assessment (MOCA) for testing of cognitive function [28].

### Assessment of olfaction

Olfaction was assessed in all RBD patients, but not in PD patients and controls, by means of the standardized Sniffin’ Sticks test as described previously [33]. The bilateral olfactory threshold, odour identification and odour discrimination were investigated with three separate subtests. The scores of threshold (T), discrimination (D) and identification (I) were summed and the combined value (TDI) categorized into five defined stages: anosmia (TDI score ≤15), severe hyposmia (16–20), moderate hyposmia (21–25), mild hyposmia (26–30), and normosmia (TDI >30).

## ^123^I-FP-CIT SPECT

In all RBD patients and 11 PD patients the presynaptic density of nigrostriatal terminals was visualized and quantified by SPECT and the dopamine transporter tracer N-ω-fluoropropyl-2β-carbomethoxy-3β-(4-iodophenyl)tropane ([^123^I]FP-CIT). Before injection of 185 MBq [^123^I]FP-CIT, the thyroid gland was blocked with sodium perchlorate. Scans were acquired on a dual-headed gamma camera (RBD patients: Symbia S; PD patients: Symbia T2; Siemens, Erlangen, Germany) 180 min after injection, a time-point at which the specific striatal to non-specific binding ratios are stable for several hours [4]. The images were obtained in 120 projections over a 360° arc by means of the step-and-shoot mode with a 40 s acquisition time per projection. Transverse reconstructed slices were generated as previously reported [40].

All scans, apart from one where no raw data were available, were analysed with Brain Analysis Software (BRASS version PDR 2.5; Hermes Medical Solutions, Sweden) (*n* = 28; 17 RBD, 11 PD). The RBD patient for whom no raw data were available showed a reduction of striatal dopamine transporter binding, particularly in the putamen, but the data were not included in the calculation of the correlation between FP-CIT-SPECT data and dermal p-alpha-syn deposition due to a lack of comparability. In the Brain Analysis Software, the individual patient images were automatically coregistered to a template generated from [^123^I]FP-CIT scans obtained in healthy controls from the ENC-DAT study [38]. On this template, volumes of interest (VOIs) were defined for each caudate nucleus and each putamen bilaterally, and a large VOI for the occipital cortex (reference region). For each individual, average counts (total binding) for all voxels within the VOIs were calculated. The specific to non-specific binding ratio was used as the outcome measure and calculated as follows: (total binding in right caudate and left caudate nucleus or right putamen and left putamen − non-specific binding)/non-specific binding. Since the striatal [^123^I]FP-CIT binding declines by natural ageing, all values were corrected for age. For each study participant an age-corrected ratio lower than two SD from the mean of controls [*n* = 24; age-range 18–73 years, obtained from the ENC-DAT study (see above)] was defined as an abnormal, i.e. reduced specific to non-specific binding ratio. The lowest normal (cut-off)value was determined to be 2. For quantitative comparison and correlation analysis, the lowest putaminal value was used.

### Likelihood of prodromal PD

The MDS has recently proposed a method to calculate the likelihood of prodromal PD [2]. In this hypothetical concept, the authors selected risk markers and prodromal markers for PD based on a literature review. The likelihood ratio (LR) of each of these markers is calculated from its sensitivity and specificity as described in the literature. The total LR of a patient is obtained by multiplying the LR of all available risk and prodromal markers. The post-test probability can be calculated based on the age-dependent prior probability of each patient to develop PD and the total LR. The criteria of prodromal PD are based on the age-dependent prior probability and the total LR of a patient. For all RBD patients in our study, the total LR and the post-test probability were calculated, and we checked if the criteria of prodromal PD were fulfilled or not.

### Skin biopsy and immunofluorescence

Skin biopsies with a diameter of 5 mm were taken from four biopsy sites per person, namely from the back (C7 and Th10, paravertebral skin) and from the proximal (10 cm below the trochanter) and distal leg (10 cm above the lateral malleolus), under local anaesthesia as previously described [8]. The biopsy was taken from the right side in all patients and controls except for two RBD patients and one control who had the biopsy on the left side. No side effects of the biopsy procedure were reported. All samples were fixed with 4% paraformaldehyde for 30 min and cryopreserved until use. 50 serial sections of 20-µm thickness were cut from each biopsy and every tenth section was analysed. Thus, a total number of five sections per biopsy site and 20 sections per patient were evaluated. Section thickness was chosen based on previous studies showing that thicker sections resulted in a weaker staining. Double-immunofluorescence with antibodies to PGP9.5 (axonal marker, Zytomed Systems, Berlin, Germany, 1:1000) and to alpha-synuclein phosphorylated at Serine 129 (clone P-Syn/81A, Covance, Princeton, New Jersey, USA, 1:500) and appropriate AlexaFluor488- and Cy3-conjugated secondary antibodies was performed. The sections were evaluated using a fluorescence microscope (Ax10, Zeiss, Oberkochen, Germany) with a CARVII-system and Visiview software (Visitron GmbH, Puchheim, Germany). Images were digitized using the confocal microscope Olympus IX81 (Olympus, Tokyo, Japan) with Fluoview Olympus Software FV10-ASW4.1. Maximum projections of z stacks were performed using Image J. Biopsies were classified as positive if at least one dermal nerve fibre was p-alpha-syn-immunoreactive. Patients were considered as positive if at least one biopsy site showed p-alpha-syn-immunoreactive nerve fibres. The number of positive biopsy sites per patient was recorded. Additionally, the number of dermal autonomic structures (sweat glands, blood vessels, erector pilorum muscles) and dermal nerve bundles per biopsy was counted and the percentage of structures innervated by p-alpha-syn-positive nerve fibres was calculated as previously described [7]. The average of all four biopsy sites was used for group comparisons and correlation analysis. Evaluation of sections was performed under blinded conditions, as the examiner had no information about the underlying diagnosis (RBD, PD or control), the result of the FP-CIT-SPECT, or of the olfactory function test.

### Statistical evaluation

SPSS Statistics 23 software (IBM, Ehningen, Germany) was used for statistical analysis, GPower 3.1 (University of Düsseldorf, Germany) was used for sample size calculation. Sample size calculation was based on the primary hypothesis that p-alpha-syn is more often detectable in patients with RBD compared to normal controls. A ratio of proportions of positive cases of 45 to 1 was assumed as effect size based on previous studies on p-alpha-syn in PD and the assumption that about 80% of all RBD patients convert to PD [8]. For the application of two-sided Fisher’s exact test with a significance level of 0.05 and a power of 0.9, a sample size of at least 17 for each group was considered necessary. Numerical data were tested for normal distribution using the Kolmogorov–Smirnov test and turned out not to be normally distributed. Therefore, for comparison of numerical data the two-tailed non-parametric Kruskal–Wallis test and Mann–Whitney *U* test were used. Categorical data were tested by two-sided Fisher’s exact test. Two-sided Spearman’s rank correlation test was used for correlation analysis. A nominal significance level of 5% was applied. As this is an exploratory study, to avoid a decrease of power and missing of possibly important findings, we did not adjust for multiple comparisons [30].

## Results

### Patients

Demographic data and symptom scores of all patients and controls are summarized in Table 1 and in the Supplemental Table. Age did not differ between groups, there were more females in the control than in the RBD group (*p* = 0.009), but gender distribution did not significantly differ between RBD and PD patients.

**Table 1:** Overview of demographic data, clinical scores and p-alpha-syn deposition (number of sites and percentage of structures positive for p-alpha-syn) of patients with RBD, PD and normal controls

	RBD with normal FP-CIT-SPECT	RBD with pathological FP-CIT-SPECT	PD	Healthy controls
Number (*n*), gender (male/female)	8; 6 male, 2 female	10; 9 male, 1 female	25; 14 male, 11 female	20; 8 male, 12 female
Median age (years) (quartiles)	62 (57.75/69.25)	65 (59.5/66.75)	63 (59/67)	59 (52/69)
Median RBD-SQ (quartiles)	10 (8.75/10.5)	10 (7.5/10)	–	–
Median MoCA (quartiles)	28 (26.5/29.5)	26 (24.75/28.25)	–	–
Median BDI (quartiles)	16 (12.75/20.25)	8·5 (2.75/17.5)	–	–
Patients with p-alpha-syn deposition	3 (37.5%)	7 (70%)	20 (80%)	0
Median percentage of p-alpha-syn-positive structures (quartiles)	0 (0/6.10)	2.25 (0.15/6.16)	3.67 (1.63/7.00)	0
Median number of p-alpha-syn-positive biopsy sites (quartiles)	0 (0/2)	1 (0.25/2.5)	2 (1/3)	0
Median duration of RBD (years) (quartiles)	3.5 (1.95/6.25)	5.5 (3.75/7.75)	–	–
Median UPDRS 1 (quartiles)	3 (1/5.75)	2.5 (1.75/4)	4.5 (4/8)	–
Median UPDRS 2 (quartiles)	1 (0.25/2.5)	0 (0/1)	4 (3/4)	–
Median UPDRS 3 (quartiles)	2.5 (0.75/4.75)	1 (0/2.75)	19 (11.75/21.25)	–
Median NMS (quartiles)	10 (7.5/11.75)	8 (4.5/12.5)	28 (14/33)	–
Median TDI (quartiles)	23.88 (14.88/30.25)	12 (6/23.94)	–	–

*BDI* Beck Depression Inventory, *FP-CIT-SPECT* ^123^I-2beta-carbomethoxy-3beta-(4-iodophenyl)-N-(3-fluoropropyl)-nortropane Single photon emission computed tomography, NMS non-motor symptom score, *p*-*alpha*-*syn* phosphorylated alpha-synuclein, *PD* Parkinson’s disease, *RBD* REM sleep behaviour disorder, *RBD*-*SQ* RBD screening questionnaire,* UPDRS* Unified Parkinson’s Disease Rating Scale

### Detection of p-alpha-syn in dermal nerve fibres in RBD

**Fig. 1:**
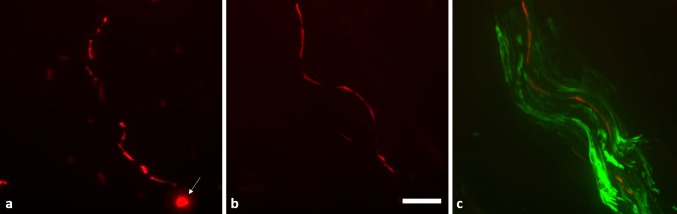
Immunofluorescence staining with anti-p-alpha-syn (*red*) and anti-PGP9.5 (*green*, ** c**). Dermal p-alpha-syn deposition was found within axons of somatosensory or autonomic nerve fibres (**b**) and resembled Lewy neurites that are typically found in the CNS (**a**). Lewy bodies as seen in CNS tissue (**a**, *arrow*) are not detectable in skin biopsies as the cell bodies of somatosensory neurons are located in the dorsal root ganglia and sympathetic ganglia. P-alpha-syn immunoreactivity forms a punctuated line within the nerve fibres. **c** illustrates the localization of p-alpha-syn deposition within a nerve fibre of a dermal nerve bundle. *Scale bar* 10 µm *p-alpha-syn* phosphorylated alpha-synuclein

**Fig. 2:**
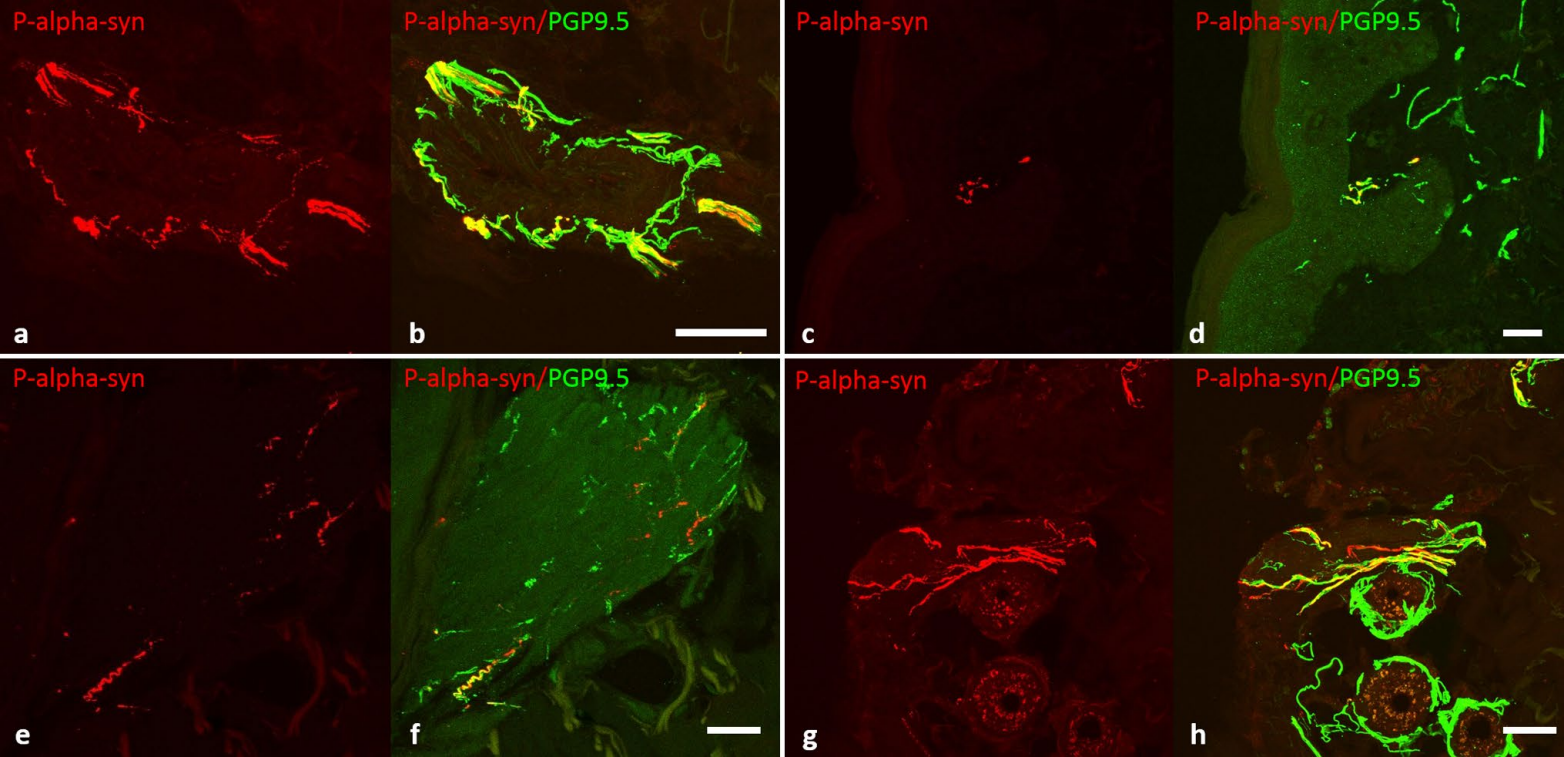
Confocal photomicrographs of double-immunofluorescence with anti-p-alpha-syn (red) and anti-PGP 9.5 (axonal marker, green). P-alpha-syn -immunoreactive nerve fibres can be found in vasomotor nerve fibres around vessels (**a**, **b**), in somatosensory nerve fibres of the subepidermal plexus (**c**, **d**), in pilomotor nerve fibres of erector pilorum muscles (**e**, **f**) and in sudomotor fibres around sweat glands (**g**, **h**). *Scale bar* 20 µm. *p*-*alpha*-*syn* phosphorylated alpha-synuclein

P-alpha-syn was detected in dermal nerve fibres of 10/18 (55.6%) patients with RBD, in 9/13 patients (69.2%) with PD H&Y stage I, and in 11/12 patients (91.7%) with PD H&Y stage II, but not in any of 20 normal controls (Figs. 1, 2). P-alpha-syn immunoreactivity was found within dermal nerve fibres, resembling Lewy neurites that are known from brain tissue (Fig. 1). Thus, p-alpha-syn was detected more frequently in patients with RBD or PD compared to controls (RBD vs controls: *p* = 0.0001, PD vs controls: *p* = 0.0001) with a sensitivity of 55.6% in RBD and 80% in PD and a specificity of 100%. The median number of p-alpha-syn deposits and the median percentage of dermal structures innervated by p-alpha-syn-positive fibres are given in Table 1. Most p-alpha-syn-positive dermal nerve fibres were autonomic fibres around blood vessels (six RBD patients, 18 PD patients, Fig. 2a, b), erector pilorum muscles (one RBD patient, four PD patients, Fig. 2e, f) or sweat glands (two RBD patients, six PD patients, Fig. 2g, h). Fibres within dermal nerve bundles not attached to a certain autonomic structure were affected in all ten p-alpha-syn-positive RBD patients and 12 PD patients. P-alpha-syn was found in somatosensory nerve fibres of the subepidermal plexus in four patients with RBD and in three patients of the PD group (Fig. 2c, d) (Supplemental Table). In two patients with RBD only somatosensory nerve fibres were affected, in two RBD patients, autonomic and somatosensory fibres were positive. In all three patients with PD and involvement of somatosensory subepidermal nerve fibres, autonomic fibres were involved as well. In summary, overall fibre type involvement in RBD was similar to PD.

In the ten p-alpha-syn-positive RBD patients, most p-alpha-syn-positive nerve fibres were found in biopsies of the neck and back (C7, five patients; Th10, eight patients), fewer in the legs (proximal leg: three patients, distal leg: five patients). In the 20 p-alpha-syn-positive PD patients, there was a trend of more p-alpha-syn-positive fibres to be found in the periphery (distal leg, 12 patients; proximal leg, 11 patients; Th10, 10 patients and C7, 8 patients); however, the differences of affected biopsy sites were not significant (Supplemental Table).

### Negative correlation between dopamine transporter binding and dermal p-alpha-syn deposition

**Fig. 3:**
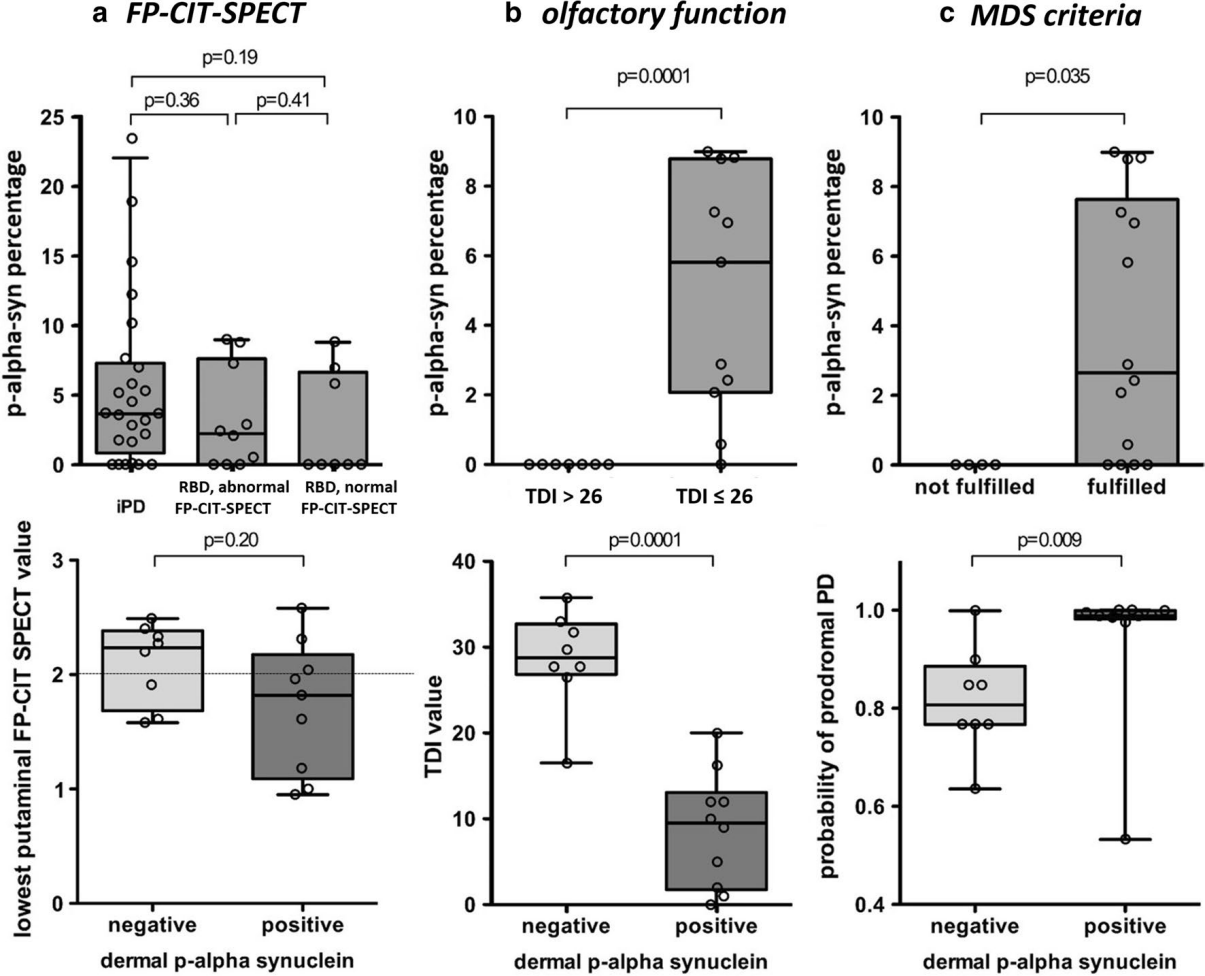
*Box plots * illustrating skin biopsy, FP-CIT-SPECT, LR and TDI data. The* box plots* of the upper panels **a**–**c** illustrate the median percentage of p-alpha-syn-positive dermal nerve structures per patient (*y*-axis) in patients with abnormal/normal FP-CIT-SPECT (**a**), normosmic (TDI > 26)/hyposmic (TDI ≤ 26) patients (**b**) and patients fulfilling/not fulfilling the research MDS criteria of prodromal PD (*x*-axis) (**c**). In **a**, upper panel, PD patients are included, all other panels only represent the RBD group. The *black line* marks the median, the box represents the quartiles, the whiskers mark the range. Individual patient values are represented by *circles*. The percentage of p-alpha-syn-positive dermal structures shows a trend towards higher numbers in PD, declining from PD to RBD with abnormal, FP-CIT-SPECT (i.e. reduced specific to non-specific binding ratio) and further to RBD with normal FP-CIT-SPECT (**a**). P-alpha-syn deposition is not found in normosmic patients, but is high in hyposmic patients (**b**) and is much more abundant in patients fulfilling the MDS criteria of prodromal PD (**c**). The * box plots* of the lower images **a**–**c** compare the lowest putaminal FP-CIT-SPECT values, TDI values and the probability of prodromal PD (*y*-axis) in patients without (negative) and with (positive) p-alpha-syn deposition in the skin biopsy (*x*-axis). Putaminal FP-CIT-SPECT values tend to be lower in patients with at least one p-alpha-syn deposition (positive) vs in patients with no deposition (negative) (**a**). In **a** the punctate line indicates the lower normal value (2.0) of the FP-CIT-SPECT value. TDI values (**b**) are significantly lower in patients with p-alpha-syn deposits in the skin biopsy and the probability of prodromal PD is higher in patients with p-alpha-syn deposition (**c**). In **a**, lower panel, only 17 individual patient values are represented by *circles* as the FP-CIT-SPECT of one patient was not included into the quantitative analysis due to lack of raw data. *FP*-*CIT*-*SPECT* ^123^I-2beta-carbomethoxy-3beta-(4-iodophenyl)-N-(3-fluoropropyl)-nortropane single photon emission computed tomography, *p*-*alpha*-*syn* phosphorylated alpha-synuclein, *PD* Parkinson’s disease, *RBD* REM sleep behaviour disorder, *TDI* olfactory threshold, discrimination and identification score

FP-CIT-SPECT was performed in all 18 patients with RBD and in 11 patients with PD and revealed diminished striatal dopamine transporter binding in 10/18 patients with RBD and in all patients with PD (Fig. 3a). Eight patients with RBD showed normal striatal dopamine transporter binding ratios above the cut-off value of two in the left and right putamen. Of the ten RBD patients with diminished dopamine transporter binding seven showed deposition of p-alpha-syn in the skin biopsy (Fig. 3a). On the other hand, seven of ten RBD patients with p-alpha-syn in the skin biopsy showed a reduced striatal dopamine transporter binding, whereas three of the p-alpha-syn-positive RBD patients had a normal FP-CIT-SPECT. Of these three patients, one patient’s dopamine transporter binding in the putamen was just above the cut-off value of 2 (2.04), the other patients showed a dopamine transporter binding of 2.58 and 2.31 (Fig. 3a, lower panel).

The median percentage of dermal structures innervated by p-alpha-syn-positive fibres was not significantly different in patients with PD compared to RBD patients with normal or pathological FP-CIT-SPECT. However, there was a trend to a higher percentage in iPD (3.67) than in RBD with (2.25) and without (0) pathological FP-CIT-SPECT (Fig. 3a, upper panel).

The lowest putaminal value of the FP-CIT-SPECT in the RBD patients (*n* = 17; one patient without raw data, thus not included in this analysis) was inversely correlated with the percentage of dermal structures innervated by p-alpha-syn-positive nerve fibres (*ρ* = −0.38, *p* = 0.048) and the number of biopsy sites with p-alpha-syn deposition (*ρ* = −0.40, *p* = 0.037). The lowest putaminal FP-CIT-SPECT value tended to be lower in patients with p-alpha-syn deposition compared to patients without p-alpha-syn deposition in the skin biopsy, but the difference failed to reach significance (*p* = 0.2, Fig. 3a, lower panel).

### Negative correlation between olfactory function and dermal p-alpha-syn deposition in RBD

Olfactory function was evaluated in all patients with RBD, resulting in a median TDI of 16.4 (range 0–35.75). Olfactory dysfunction could be diagnosed in 15/18 patients (83·3%), further categorized as anosmia (TDI <15) in eight patients, severe hyposmia (TDI 16–20) in three patients, mild hyposmia (TDI 26–30) in four patients. All eight patients with anosmia and two of three patients with severe hyposmia displayed p-alpha-syn deposition in the skin biopsies; however, there was no p-alpha-syn deposition in the four patients with mild hyposmia or the three patients with normal olfactory function. P-alpha-syn deposition was inversely correlated with olfactory function (number of positive biopsy sites and TDI: rho = −0.64 *p* = 0.004, percentage of positive dermal structures and TDI: rho = −0.67, *p* = 0.002). The number of p-alpha-syn-positive biopsy sites and the percentage of p-alpha-syn-positive structures was higher in anosmic, severe or moderate hyposmic RBD patients compared to mildly hyposmic or normosmic patients (*p* = 0.0001, Fig. 3b, upper panel), and patients with p-alpha-syn deposition in the skin biopsy had lower TDI values (*p* = 0.0001, Fig. 3b, lower panel).

### Positive correlation between p-alpha-syn deposition and likelihood of prodromal PD

Based on the research criteria of the MDS, the LR of 14 of 18 RBD patients fulfilled the criteria for prodromal PD [2]. Dermal p-alpha-syn was found in ten patients who fulfilled the criteria and not in the four patients whose LR was below the age-dependent cut-off value. The LR and the post-test probabilities of prodromal PD correlated with the number of positive biopsy sites (total LR: *ρ* = 0.51, *p* = 0.032, post-test probability: *ρ* = 0.55, *p* = 0.019) and the percentage of dermal structures with p-alpha-syn-positive fibres (total LR: *ρ* = 0.53, *p* = 0.023, post-test probability: *ρ* = 0.57, *p* = 0.019). The percentage of p-alpha-syn-positive dermal structures and the number of p-alpha-syn-positive biopsy sites was higher in patients who fulfilled the research criteria for prodromal PD (*p* = 0.035, Fig. 3c) and p-alpha-syn-positive RBD patients had a post-test probability of (*p* = 0.009, Fig. 3c, lower panel).

## Discussion

The present study provides evidence of p-alpha-syn deposition in dermal nerve fibres of patients with RBD, which is often a prodromal stage of a synucleinopathy, mostly PD or DLB. We show a high probability (10/11 patients with moderate or severe hyposmia) of dermal p-alpha-syn deposition in RBD patients with olfactory dysfunction, most of whom also had reduced dopamine transporter binding in FP-CIT-SPECT. Our data encourage the use of skin biopsy as a histopathological stratification tool for clinical studies in RBD and PD patients.

Identification of patients with prodromal PD is of high interest for clinical studies with disease-modifying drugs. Patients with RBD carry a high risk to convert to PD, and therefore comprise a patient group of special interest. According to present knowledge, up to 15% of RBD patients will not convert to an alpha-synucleinopathy within 15 years [16, 18, 23]. Therefore, stratification tools are necessary to identify those RBD patients who suffer from an alpha-synucleinopathy as indicated by p-alpha-syn deposition and therefore have the highest risk of manifesting motor symptoms. For this purpose, probability criteria, including FP-CIT-SPECT, RBD and hyposmia, were introduced by the MDS and are currently validated [2, 21]. The present data show a correlation of dermal p-alpha-syn deposition not only with single items of these criteria like reduced dopamine transporter binding in the FP-CIT-SPECT and hyposmia, but also with the probability score in total. Thus, p-alpha-syn deposition in dermal nerve fibres may be a suitable stratification marker for prodromal PD. Additionally, in contrast to FP-CIT-SPECT and other potential measures of prodromal PD, skin biopsy provides direct histopathological evidence of p-alpha-syn and may therefore be of particular interest in clinical studies targeting p-alpha-syn deposition. Patients combining abnormal FP-CIT-SPECT and p-alpha-syn deposition in the skin may represent a group of even higher interest for these clinical trials.

In RBD, dermal p-alpha-syn deposition was detected with a frequency of 55.6% and correlated with other risk markers of PD development as displayed by the total LR and the post-test probability of prodromal PD as proposed by the MDS. It needs to be emphasized that RBD is included in the calculation of the total LR. For a general conclusion on the association between prodromal PD according to these research criteria and p-alpha-syn deposition, studies including cases of prodromal PD without RBD would be needed.

The sensitivity of p-alpha-syn detection in skin biopsies of RBD patients may be influenced by the disease stage of RBD: our cohort comprises a relatively large percentage of RBD patients with abnormal FP-CIT-SPECT indicating the conversion to motor PD. Larger, preferentially multicentre studies are needed to determine the sensitivity of dermal p-alpha-syn deposition in RBD patients of different stages of disease. Our data show that dermal p-alpha-syn can be detected prior to the onset of motor symptoms and may indicate a high risk of conversion to motor PD. Hyposmia is supposed to occur approximately 5 years before conversion of patients with RBD to motor PD [29], and FP-CIT-SPECT is considered a progressive marker for the risk of conversion of patients with RBD to PD [19]. To determine the exact time-point of dermal p-alpha-syn deposition in relation to the onset of manifest PD will require longitudinal studies. In our investigated cohort of RBD patients, p-alpha-syn deposition was present in almost all patients with severe hyposmia and in most patients with reduced dopamine transporter binding in the FP-CIT-SPECT. In three patients, however, the deposition of p-alpha-syn in the skin was associated with a normal FP-CIT-SPECT, i.e. it occurred before the development of a premotor dopaminergic nigrostriatal dysfunction. Whether a higher proportion of RBD patients with a normal FP-CIT-SPECT than in our cohort already have dermal p-alpha-syn deposits remains to be demonstrated. If this were the case, dermal p-alpha-syn deposits might be an earlier indicator of future conversion to clinical PD than FP-CIT-SPECT. On the other hand, RBD patients with a normal FP-CIT-SPECT, normosmia, and a negative finding for p-alpha-syn in skin biopsy (*n* = 6) may either represent a stage where conversion into PD is not yet detectable, or may belong to the approximately 15% RBD patients who will not convert. In contrast to PD where no stage-dependent differences of p-alpha-syn deposition was found in previous studies, our data show that p-alpha-syn deposition increases with advanced stages of RBD as represented by olfactory dysfunction and abnormal FP-CIT-SPECT. A testable hypothesis would be that dermal p-alpha-syn spreading occurs during the course of RBD and does not further increase at the stage of motor PD.

By taking biopsies from four different sites and performing serial sections, we achieved a moderate sensitivity and high specificity of p-alpha-syn detection as a biomarker for PD in patients with early PD (Hoehn and Yahr stage I–II; sensitivity of 80%, specificity of 100%). Sensitivity and specificity of p-alpha-syn as a biomarker of PD at the onset of motor symptoms does not appear to differ from later stages of the disease that were assessed in previous studies [6, 8]. However, sensitivity and specificity differs between studies [6, 8, 10, 12, 26, 41], presumably due to different protocols, biopsy sites and number of sections analysed [9]. We show that serial cryosections and multiple biopsy sites increase sensitivity. Detailed comparative methodological studies need to be performed to develop the best possible protocol for this method. In contrast to previous studies, we could not find a significant decrease of p-alpha-syn deposition from proximal to distal biopsy sites; however, the sample size is too low to draw any conclusion from that.

Consistent with an early involvement of the peripheral autonomic system in PD, p-alpha-syn deposition has recently been reported in gastric and enteric biopsies of premotor PD and in colon biopsies of patients with RBD [32, 35]. Results were inconsistent with moderate sensitivity and low specificity in the study on prodromal PD [35] and low sensitivity but high specificity in the study on RBD patients [32], thus not allowing any conclusion on the usefulness of enteric biopsies as a stratification tool for RBD patients. A recent study evaluating p-alpha-syn deposition in submandibular salivary gland biopsies of RBD patients showed a promising high sensitivity and specificity of p-alpha-syn detection, but also had a procedural limitation as less than half of the biopsies of the RBD patients contained glandular parenchyma [39]. In comparison with enteric biopsies and submandibular gland biopsy, skin biopsy appears to be a reasonable stratification tool to identify RBD patients with a high risk of conversion to PD, as it is technically easy to perform and provides a high specificity.

As in former studies, we found p-alpha-syn deposits mainly in autonomic fibres in patients with RBD [6, 8]. Similarly, in PD unmyelinated somatosensory dermal nerve fibres harbour p-alpha-syn deposits less frequently than autonomic fibres [6, 8, 10]. In contrast, somatosensory fibres were the major fibre type affected in patients with multiple system atrophy [10]. The detection of p-alpha-syn in somatosensory dermal nerve fibres in three patients with RBD in the present study provides evidence that somatosensory nerve fibres are also involved at premotor stages.

A limitation of our study is the lack of longitudinal data. A major question that remains to be answered is the outcome of the eight patients who did not harbour p-alpha-syn deposition in their skin biopsies. Three out of these eight patients had a reduced nigrostriatal dopamine transporter ligand binding in FP-CIT-SPECT, whereas the remaining five patients presented a normal FP-CIT-SPECT (Fig. 3a, lower panel). Whether the three patients with an already affected nigrostriatal dopamine system or all of the eight patients will develop PD/DLB or multiple system atrophy or whether some or all of them belong to the small percentage of patients with RBD who do not develop a synucleinopathy will be the subject of longitudinal studies that need to follow. Large multicentre longitudinal studies also need to be performed to study the temporal relationship between dermal p-alpha-syn deposition and conversion to PD.

In summary, our study provides evidence that phosphorylated alpha-synuclein deposition is present in dermal nerve fibres in a subgroup of patients suffering from RBD. Considering its relation to other risk factors for conversion from RBD to PD such as hyposmia and reduced FP-CIT dopamine transporter uptake, p-alpha-syn deposition in skin may be used in RBD patients as a peripheral histopathological marker of an alpha-synucleinopathy, prior to the onset of PD motor symptoms. Given the simple access to skin biopsy and its high specificity, the method may assist in future stratification of RBD patient cohorts for clinical trials testing disease-modifying drugs.

## Electronic supplementary material

Below is the link to the electronic supplementary material.

Supplementary material 1 (DOCX 19 kb)

## References

[1] Beck A, Steer R, Brown G (1996). The Beck depression inventory-second edition manual.

[2] Berg D, Postuma RB, Adler CH, Bloem BR, Chan P, Dubois B, Gasser T, Goetz CG, Halliday G, Joseph L, Lang AE, Liepelt-Scarfone I, Litvan I, Marek K, Obeso J, Oertel W, Olanow CW, Poewe W, Stern M, Deuschl G (2015). MDS research criteria for prodromal Parkinson’s disease. Mov Disord.

[3] Boeve BF, Silber MH, Ferman TJ, Lin SC, Benarroch EE, Schmeichel AM, Ahlskog JE, Caselli RJ, Jacobson S, Sabbagh M, Adler C, Woodruff B, Beach TG, Iranzo A, Gelpi E, Santamaria J, Tolosa E, Singer C, Mash DC, Luca C, Arnulf I, Duyckaerts C, Schenck CH, Mahowald MW, Dauvilliers Y, Graff-Radford NR, Wszolek ZK, Parisi JE, Dugger B, Murray ME, Dickson DW (2013). Clinicopathologic correlations in 172 cases of rapid eye movement sleep behavior disorder with or without a coexisting neurologic disorder. Sleep Med.

[4] Booij J, Hemelaar TG, Speelman JD, de Bruin K, Janssen AG, van Royen EA (1999). One-day protocol for imaging of the nigrostriatal dopaminergic pathway in Parkinson’s disease by [123I]FPCIT SPECT. J Nucl Med.

[5] Braak H, de Vos RA, Bohl J, Del Tredici K (2006). Gastric alpha-synuclein immunoreactive inclusions in Meissner’s and Auerbach’s plexuses in cases staged for Parkinson’s disease-related brain pathology. Neurosci Lett.

[6] Donadio V, Incensi A, Leta V, Giannoccaro MP, Scaglione C, Martinelli P, Capellari S, Avoni P, Baruzzi A, Liguori R (2014). Skin nerve alpha-synuclein deposits: a biomarker for idiopathic Parkinson disease. Neurology.

[7] Donadio V, Incensi A, Piccinini C, Cortelli P, Giannoccaro MP, Baruzzi A, Liguori R (2016). Skin nerve misfolded alpha-synuclein in pure autonomic failure and Parkinson disease. Ann Neurol.

[8] Doppler K, Ebert S, Uceyler N, Trenkwalder C, Ebentheuer J, Volkmann J, Sommer C (2014). Cutaneous neuropathy in Parkinson’s disease: a window into brain pathology. Acta Neuropathol.

[9] Doppler K, Volkmann J, Sommer C (2015). Skin biopsies in the differential diagnosis of parkinsonism: are we ready for simplified protocols?. Brain.

[10] Doppler K, Weis J, Karl K, Ebert S, Ebentheuer J, Trenkwalder C, Klebe S, Volkmann J, Sommer C (2015). Distinctive distribution of phospho-alpha-synuclein in dermal nerves in multiple system atrophy. Mov Disord.

[11] Fearnley JM, Lees AJ (1991). Ageing and Parkinson’s disease: substantia nigra regional selectivity. Brain.

[12] Gibbons CH, Garcia J, Wang N, Shih LC, Freeman R (2016). The diagnostic discrimination of cutaneous alpha-synuclein deposition in Parkinson disease. Neurology.

[13] Goetz CG, Tilley BC, Shaftman SR, Stebbins GT, Fahn S, Martinez-Martin P, Poewe W, Sampaio C, Stern MB, Dodel R, Dubois B, Holloway R, Jankovic J, Kulisevsky J, Lang AE, Lees A, Leurgans S, LeWitt PA, Nyenhuis D, Olanow CW, Rascol O, Schrag A, Teresi JA, van Hilten JJ, LaPelle N (2008). Movement Disorder Society-sponsored revision of the Unified Parkinson’s Disease Rating Scale (MDS-UPDRS): scale presentation and clinimetric testing results. Mov Disord.

[14] Hoehn MM, Yahr MD (1967). Parkinsonism: onset, progression and mortality. Neurology.

[15] Hughes AJ, Daniel SE, Kilford L, Lees AJ (1992). Accuracy of clinical diagnosis of idiopathic Parkinson’s disease: a clinico-pathological study of 100 cases. J Neurol Neurosurg Psychiatry.

[16] Iranzo A, Fernandez-Arcos A, Tolosa E, Serradell M, Molinuevo JL, Valldeoriola F, Gelpi E, Vilaseca I, Sanchez-Valle R, Llado A, Gaig C, Santamaria J (2014). Neurodegenerative disorder risk in idiopathic REM sleep behavior disorder: study in 174 patients. PLoS One.

[17] Iranzo A, Lomena F, Stockner H, Valldeoriola F, Vilaseca I, Salamero M, Molinuevo JL, Serradell M, Duch J, Pavia J, Gallego J, Seppi K, Hogl B, Tolosa E, Poewe W, Santamaria J (2010). Decreased striatal dopamine transporter uptake and substantia nigra hyperechogenicity as risk markers of synucleinopathy in patients with idiopathic rapid-eye-movement sleep behaviour disorder: a prospective study [corrected]. Lancet Neurol.

[18] Iranzo A, Tolosa E, Gelpi E, Molinuevo JL, Valldeoriola F, Serradell M, Sanchez-Valle R, Vilaseca I, Lomena F, Vilas D, Llado A, Gaig C, Santamaria J (2013). Neurodegenerative disease status and post-mortem pathology in idiopathic rapid-eye-movement sleep behaviour disorder: an observational cohort study. Lancet Neurol.

[19] Iranzo A, Valldeoriola F, Lomena F, Molinuevo JL, Serradell M, Salamero M, Cot A, Ros D, Pavia J, Santamaria J, Tolosa E (2011). Serial dopamine transporter imaging of nigrostriatal function in patients with idiopathic rapid-eye-movement sleep behaviour disorder: a prospective study. Lancet Neurol.

[20] Lebouvier T, Neunlist M, Varannes SB, Coron E, Drouard A, N’Guyen JM, Chaumette T, Tasselli M, Paillusson S, Flamand M, Galmiche JP, Damier P, Derkinderen P (2010). Colonic biopsies to assess the neuropathology of Parkinson’s disease and its relationship with symptoms. PLoS One.

[21] Mahlknecht P, Gasperi A, Willeit P, Kiechl S, Stockner H, Willeit J, Rungger G, Sawires M, Nocker M, Rastner V, Mair KJ, Hotter A, Poewe W, Seppi K (2016). Prodromal Parkinson’s disease as defined per MDS research criteria in the general elderly community. Mov Disord.

[22] Mahlknecht P, Iranzo A, Hogl B, Frauscher B, Muller C, Santamaria J, Tolosa E, Serradell M, Mitterling T, Gschliesser V, Goebel G, Brugger F, Scherfler C, Poewe W, Seppi K (2015). Olfactory dysfunction predicts early transition to a Lewy body disease in idiopathic RBD. Neurology.

[23] Mahowald MW, Schenck CH (2013). REM sleep behaviour disorder: a marker of synucleinopathy. Lancet Neurol.

[24] Marek K, Innis R, van Dyck C, Fussell B, Early M, Eberly S, Oakes D, Seibyl J (2001). [123I]beta-CIT SPECT imaging assessment of the rate of Parkinson’s disease progression. Neurology.

[25] American Academy of Sleep Medicine. International Classification of Sleep Disorders: Diagnostic and Coding Manual. In: Medicine. AAoS (ed). 2 edn. Westchester III

[26] Miki Y, Tomiyama M, Ueno T, Haga R, Nishijima H, Suzuki C, Mori F, Kaimori M, Baba M, Wakabayashi K (2010). Clinical availability of skin biopsy in the diagnosis of Parkinson’s disease. Neurosci Lett.

[27] Morrish PK, Sawle GV, Brooks DJ (1995). Clinical and [18F] dopa PET findings in early Parkinson’s disease. J Neurol Neurosurg Psychiatry.

[28] Nasreddine ZS, Phillips NA, Bedirian V, Charbonneau S, Whitehead V, Collin I, Cummings JL, Chertkow H (2005). The Montreal Cognitive Assessment, MoCA: a brief screening tool for mild cognitive impairment. J Am Geriatr Soc.

[29] Postuma RB, Gagnon JF, Vendette M, Desjardins C, Montplaisir JY (2011). Olfaction and color vision identify impending neurodegeneration in rapid eye movement sleep behavior disorder. Ann Neurol.

[30] Rothman KJ (1990). No adjustments are needed for multiple comparisons. Epidemiology.

[31] Schenck CH, Montplaisir JY, Frauscher B, Hogl B, Gagnon JF, Postuma R, Sonka K, Jennum P, Partinen M, Arnulf I, Cochen de Cock V, Dauvilliers Y, Luppi PH, Heidbreder A, Mayer G, Sixel-Doring F, Trenkwalder C, Unger M, Young P, Wing YK, Ferini-Strambi L, Ferri R, Plazzi G, Zucconi M, Inoue Y, Iranzo A, Santamaria J, Bassetti C, Moller JC, Boeve BF, Lai YY, Pavlova M, Saper C, Schmidt P, Siegel JM, Singer C, St Louis E, Videnovic A, Oertel W (2013). Rapid eye movement sleep behavior disorder: devising controlled active treatment studies for symptomatic and neuroprotective therapy—a consensus statement from the International Rapid Eye Movement Sleep Behavior Disorder Study Group. Sleep Med.

[32] Sprenger FS, Stefanova N, Gelpi E, Seppi K, Navarro-Otano J, Offner F, Vilas D, Valldeoriola F, Pont-Sunyer C, Aldecoa I, Gaig C, Gines A, Cuatrecasas M, Hogl B, Frauscher B, Iranzo A, Wenning GK, Vogel W, Tolosa E, Poewe W (2015). Enteric nervous system alpha-synuclein immunoreactivity in idiopathic REM sleep behavior disorder. Neurology.

[33] Stiasny-Kolster K, Doerr Y, Moller JC, Hoffken H, Behr TM, Oertel WH, Mayer G (2005). Combination of ‘idiopathic’ REM sleep behaviour disorder and olfactory dysfunction as possible indicator for alpha-synucleinopathy demonstrated by dopamine transporter FP-CIT-SPECT. Brain.

[34] Stiasny-Kolster K, Mayer G, Schafer S, Moller JC, Heinzel-Gutenbrunner M, Oertel WH (2007). The REM sleep behavior disorder screening questionnaire–a new diagnostic instrument. Mov Disord.

[35] Stokholm MG, Danielsen EH, Hamilton-Dutoit SJ, Borghammer P (2016). Pathological alpha-synuclein in gastrointestinal tissues from prodromal Parkinson disease patients. Ann Neurol.

[36] Storch A, Odin P, Trender-Gerhard I, Fuchs G, Reifschneider G, Ray Chaudhuri K, Jost WH, Ebersbach G (2010). Non-motor Symptoms Questionnaire and Scale for Parkinson’s disease. Cross-cultural adaptation into the German language. Nervenarzt.

[37] Uchiyama M, Isse K, Tanaka K, Yokota N, Hamamoto M, Aida S, Ito Y, Yoshimura M, Okawa M (1995). Incidental Lewy body disease in a patient with REM sleep behavior disorder. Neurology.

[38] Varrone A, Dickson JC, Tossici-Bolt L, Sera T, Asenbaum S, Booij J, Kapucu OL, Kluge A, Knudsen GM, Koulibaly PM, Nobili F, Pagani M, Sabri O, Vander Borght T, Van Laere K, Tatsch K (2013). European multicentre database of healthy controls for [123I]FP-CIT SPECT (ENC-DAT): age-related effects, gender differences and evaluation of different methods of analysis. Eur J Nucl Med Mol Imaging.

[39] Vilas D, Iranzo A, Tolosa E, Aldecoa I, Berenguer J, Vilaseca I, Marti C, Serradell M, Lomena F, Alos L, Gaig C, Santamaria J, Gelpi E (2016). Assessment of alpha-synuclein in submandibular glands of patients with idiopathic rapid-eye-movement sleep behaviour disorder: a case–control study. Lancet Neurol.

[40] Winz OH, Hellwig S, Mix M, Weber WA, Mottaghy FM, Schafer WM, Meyer PT (2012). Image quality and data quantification in dopamine transporter SPECT: advantage of 3-dimensional OSEM reconstruction?. Clin Nucl Med.

[41] Zange L, Noack C, Hahn K, Stenzel W, Lipp A (2015). Phosphorylated alpha-synuclein in skin nerve fibres differentiates Parkinson’s disease from multiple system atrophy. Brain.

