# Supplementary material for: Dermal phospho-alpha-synuclein deposits confirm REM sleep behaviour disorder as prodromal Parkinson’s disease
Source: Acta Neuropathol. 2017 Feb 8;133(4):535–45. doi: 10.1007/s00401-017-1684-z (PMC5348554; doi:10.1007/s00401-017-1684-z)
Supplement: Supplementary file 1 — Supplementary material 1 (DOCX 19 kb) [file 401_2017_1684_MOESM1_ESM.docx]

Supplemental Table: Summary of the data of all patients. p-alpha-syn=phospho-alpha-synuclein, UL=upper leg, LL=lower leg, m=male,f=female, ves=vessel, db=dermal nerve bundle, sg=sweat gland,subepi=subepidermal plexus, ep=erector pilorum muscle, n/a=not available, FP-CIT-SPECT=123I-N-ω-fluoropropyl-2β-carbomethoxy-3β-(4-iodophenyl)tropane Single Photon Emission Computed Tomography, RBD=Rapid eye movement sleep behaviour disorder, PD=Parkinson’s disease, RBD-SQ= RBD screening questionnaire, UPDRS= Unified Parkinson’s Disease Rating Scale, BDI= Beck Depression Inventory, TDI= threshold, discrimination and identification, LR=likelihood ratio, H&Y=Hoehn and Yahr

| patient ID | diagnosis | age/gender | p-alpha-syn-positive biopsy sites | Structure innervated by p-alpha-syn-positive fibres | number of positive biopsy sites | percentage of p-alpha-syn-positive structures (%) | lowest FP-CIT-SPECT value | RBD-SQ | UPDRS part 3 | BDI | TDI score | LR | post-test probability |
| --- | --- | --- | --- | --- | --- | --- | --- | --- | --- | --- | --- | --- | --- |
| 1 | RBD | 66/m | C7 | ves, db | 1 | 2.075 | 1 | 11 | 3 | 16 | 5 | 58919 | 0.999 |
| 2 | RBD | 59/m | - | - | 0 | 0 | 1·91 | 10 | 1 | 21 | 33 | 1677 | 0.927 |
| 3 | RBD | 57/m | - | - | 0 | 0 | 2·4 | 9 | 0 | 20 | 35.75 | 43 | 0.245 |
| 4 | RBD | 54/m | - | - | 0 | 0 | 2·49 | 10 | 1 | 36 | 31.75 | 147 | 0.371 |
| 5 | RBD | 64/m | Th10 | ves, db | 1 | 2.885 | 1·18 | 10 | 4 | 0 | 0 | 156431 | 0.999 |
| 6 | RBD | 66/m | UL, LL, Th10, C7 | ves, db, sg, subepi | 4 | 8.783 | 1·61 | 10 | 0 | 8 | 16.25 | 33039 | 0.999 |
| 7 | RBD | 50/m | UL | db | 1 | 0.581 | 1·96 | 3 | 1 | 0 | 12 | 10950 | 0.978 |
| 8 | RBD | 58/m | - | - | 0 | 0 | 2·2 | 8 | 4 | 15 | 29.75 | 452 | 0.774 |
| 9 | RBD | 60/m | - | - | 0 | 0 | 2·33 | 11 | 0 | 5 | 27.75 | 84 | 0.515 |
| 10 | RBD | 64/m | - | - | 0 | 0 | 2·27 | 6 | 2 | 12 | 16.5 | 989 | 0.926 |
| 11 | RBD | 70/f | Th10, C7 | subepi, db | 2 | 6·947 | 2·04 | 11 | 3 | 17 | 10 | 1042 | 0·964 |
| 12 | RBD | 73/m | - | - | 0 | 0 | 1·58 | 10 | 0 | 18 | 26·5 | 38139 | 0·999 |
| 13 | RBD | 67/m | Th10 | subepi, db | 1 | 2·42 | 1·82 | 7 | 4 | 9 | 1 | 3605491 | 1 |
| 14 | RBD | 61/m | LL, Th10, C7 | ves, db, sg | 3 | 7·253 | 0·95 | 9 | 0 | 5 | 12 | 17472 | 0·995 |
| 15 | RBD | 75/f | LL, Th10, C7 | ves, db | 3 | 8·824 | 2·58 | 10 | 10 | 13 | 20 | 7355 | 0·996 |
| 16 | RBD | 56/f | LL, UL, Th10 | ves, db | 3 | 8·993 | n/a | 12 | 2 | 19 | 9 | 118090 | 0·999 |
| 17 | RBD | 67/m | - | - | 0 | 0 | 1·61 | 7 | 0 | 2 | 27·75 | 11527 | 0·996 |
| 18 | RBD | 69/m | LL, Th10 | db, ep, subepi | 2 | 5·816 | 2·31 | 10 | 7 | 21 | 2 | 4746 | 0·99 |
| 19 | PD, H&Y I | 67/m | Th10, C7 | ves, db, ep | 2 | 3·182 | n/a | - | n/a | - | - | - | - |
| 20 | PD, H&Y I | 63/f | - | - | 0 | 0 | n/a | - | 6 | - | - | - | - |
| 21 | PD, H&Y I | 69/m | - | - | 0 | 0 | 1·78 | - | 21 | - | - | - | - |
| 22 | PD, H&Y I | 62/f | LL, UL | ves | 2 | 6·997 | n/a | - | 28 | - | - | - | - |
| 23 | PD, H&Y I | 62/m | LL, UL, Th10, C7 | ves, db, sg | 4 | 14·561 | n/a | - | 19 | - | - | - | - |
| 24 | PD, H&Y I | 44/m | C7 | db | 1 | 3·671 | n/a | - | 13 | - | - | - | - |
| 25 | PD, H&Y I | 61/m | LL | ves | 1 | 3·704 | n/a | - | 4 | - | - | - | - |
| 26 | PD, H&Y I | 73/m | LL, UL, C7 | ves, db, sg, subepi | 3 | 7·632 | 1·48 | - | 16 | - | - | - | - |
| 27 | PD, H&Y I | 73/m | LL, UL, Th10 | ves, db, sg, ep, subepi | 3 | 5·804 | 1·2 | - | 20 | - | - | - | - |
| 28 | PD, H&Y I | 54/f | - | - | 0 | 0 | 1·7 | - | 22 | - | - | - | - |
| 29 | PD, H&Y I | 59/m | UL | ves | 2 | 3·572 | 1·5 | - | 7 | - | - | - | - |
| 30 | PD, H&Y I | 63/m | - | - | 0 | 0 | 1·13 | - | 3 | - | - | - | - |
| 31 | PD, H&Y I | 67/f | Th10, C7 | ves | 2 | 23·438 | 1·23 | - | 14 | - | - | - | - |
| 32 | PD, H&Y II | 70/f | LL | ves | 1 | 2·83 | n/a | - | 9 | - | - | - | - |
| 33 | PD, H&Y II | 55/m | LL | ves | 1 | 4·518 | 1·02 | - | 11 | - | - | - | - |
| 34 | PD, H&Y II | 65/f | UL, Th10, C7 | ves, db | 3 | 5·294 | n/a | - | 19 | - | - | - | - |
| 35 | PD, H&Y II | 71/f | LL, UL | ves, db | 2 | 2·196 | n/a | - | 27 | - | - | - | - |
| 36 | PD, H&Y II | 50/f | LL, UL, Th10 | ves, db | 3 | 10·153 | 0·88 | - | 19 | - | - | - | - |
| 37 | PD, H&Y II | 67/m | LL, UL, Th10 | ves, db, ep | 3 | 12·216 | n/a | - | 30 | - | - | - | - |
| 38 | PD, H&Y II | 67/f | Th10 | ves, subepi | 1 | 1·631 | 1·51 | - | 12 | - | - | - | - |
| 39 | PD, H&Y II | 50/m | LL, UL,Th10 | ves, db | 3 | 5·16 | 1·14 | - | 23 | - | - | - | - |
| 40 | PD, H&Y II | 76/m | C7 | sg | 1 | 0·0877 | n/a | - | 19 | - | - | - | - |
| 41 | PD, H&Y II | 61/m | LL | ves, db, sg | 1 | 1·755 | n/a | - | 30 | - | - | - | - |
| 42 | PD, H&Y II | 55/m |  |  | 0 | 0 | n/a | - | 21 | - | - | - | - |
| 43 | PD, H&Y II | 63/f | UL, Th10, C7 | ves, db, ep, sg | 3 | 18·888 | n/a | - | 21 | - | - | - | - |
